# Green-Synthesized Silver Nanoparticle-Loaded Antimicrobial Films: Preparation, Characterization, and Food Preservation

**DOI:** 10.3390/foods14142509

**Published:** 2025-07-17

**Authors:** Wenxi Yu, Qin Lei, Jingxian Jiang, Jianwei Yan, Xijian Yi, Juan Cheng, Siyu Ou, Wenjia Yin, Ziyan Li, Yuru Liao

**Affiliations:** 1 School of Packaging Engineering, Hunan University of Technology, Zhuzhou 412007, China; yuwx@hut.edu.cn (W.Y.); m230805z1003@stu.hut.edu.cn (Q.L.); m210805z1001@stu.hut.edu.cn (J.J.); 13100235687@163.com (X.Y.); m24080500009@stu.hut.edu.cn (S.O.); m15580905262@163.com (Z.L.); lyr01110044@outlook.com (Y.L.); 2 National Key Laboratory of Safety and Resilience of Civil Engineering in Mountain Area, East China Jiaotong University, Nanchang 330013, China; 3 College of Packaging Engineering, Jinan University, Zhuhai 519070, China; chengj2008@jnu.edu.cn; 4 School of Packaging Design and Art, Hunan University of Technology, Zhuzhou 412007, China; m23135108093@stu.hut.edu.cn

**Keywords:** sliver nanoparticles, montmorillonite, polyvinyl alcohol, antimicrobial film, food preservation

## Abstract

This study presented a novel antimicrobial packaging PVA/xanthan gum film decorated with green-synthesized silver nanoparticles (AgNPs) derived from *Myrica rubra leaf extract* (*MRLE*) for the first time. Montmorillonite (MMT) was used to improve its dispersion (AgNPs@MMT). The synthesis time, temperature, and concentration of AgNO_3_ were considered using a central composite design coupled with response surface methodology to obtain the optimum AgNPs (2 h, 75 °C, 2 mM). Analysis of substance concentration changes confirmed that the higher phenolic and flavonoid content in *MRLE* acted as reducing agents and stabilizers in AgNP synthesis, participating in the reaction rather than adsorbing to nanoparticles. TEM, XRD, and FTIR images revealed a spherical shape of the prepared AgNPs, with an average diameter of 8.23 ± 4.27 nm. The incorporation of AgNPs@MMT significantly enhanced the mechanical properties of the films, with the elongation at break and shear strength increasing by 65.19% and 52.10%, respectively, for the PAM2 sample. The films exhibited strong antimicrobial activity against both *Escherichia coli* (18.56 mm) and *Staphylococcus aureus* (20.73 mm). The films demonstrated effective food preservation capabilities, significantly reducing weight loss and extending the shelf life of packaged grapes and bananas. Molecular dynamics simulations reveal the diffusion behavior of AgNPs in different matrices, while the measured silver migration (0.25 ± 0.03 mg/kg) complied with *EFSA* regulations (10 mg/kg), confirming its food safety. These results demonstrate the film’s potential as an active packaging material for fruit preservation.

## 1. Introduction

Foodborne diseases and high-grade food shelf life demand have drawn increasing attention to the antimicrobial properties of the new generation food packaging materials [1] since traditional ones have little effect against microbial contamination as well as its growth and reproduction [2]. Consequently, many scholars devoted themselves to prepare active food packaging materials [3] and advanced antimicrobial materials [4,5,6,7]. Among these studies, silver nanoparticles (AgNPs), owing to their good chemical stability, thermal stability, and antimicrobial ability, are widely utilized in exploring new antimicrobial materials, particularly in biomedicine and food preservation [8,9]. Extensive studies demonstrated that AgNPs can effectively inhibit bacterial growth, thus significantly prolong food shelf life [10].

Green chemistry serves as a pivotal scientific framework for sustainable development, offering essential solutions to mitigate climate change and combat environmental pollution. The green synthesis of AgNPs using plant extracts as both reducing and stabilizing agents has attracted significant attention in recent years [11,12,13], offering an eco-friendly alternative to conventional chemical synthesis methods. Muhammad et al. [12] investigated the potential of ginger extract for green synthesis of AgNPs and evaluated their antibacterial properties; the results demonstrated that the synthesized AgNPs exhibited the strongest antibacterial activity against *Escherichia coli*. Rocha et al. [14] developed an eco-friendly approach for AgNP synthesis using *Eucalyptus leaf extract* and evaluated their environmental remediation potential, demonstrating significant antibacterial activity and efficient photocatalytic dye degradation capabilities. The green synthesis method undoubtedly offers environmental and sustainability advantages; however, it also has certain limitations. For instance, the extraction process of plant extracts may require significant amounts of water, and in some cases, the availability of plant extracts may be subject to seasonal or regional constraints. Therefore, while the green synthesis of AgNPs is advantageous from an environmental perspective, addressing how to further enhance its economic feasibility and general applicability remains an important challenge that needs to be addressed. In addition, due to the poor dispersion, AgNPs has a limited application in food-contact materials as an antimicrobial agent, which drives the academic community to explore feasible strategies to disperse AgNPs and take full advantage of them [15]. Montmorillonite (MMT) has been confirmed as an excellent adsorbent for a wide range of organic [16] and inorganic substances [17]. One of the application scenarios involves its physically adsorbing ability of AgNPs, which makes it promising for enhancing their dispersibility. Moreover, it is easy to form a highly stable colloid for MMT when sprinkling AgNPs according to the interlayer constraint effect. The good structural stability brings an extra effect of significantly enhancing antimicrobial activity [18,19], which demonstrates that MMT is an ideal carrier for AgNPs.

**Figure 1:**
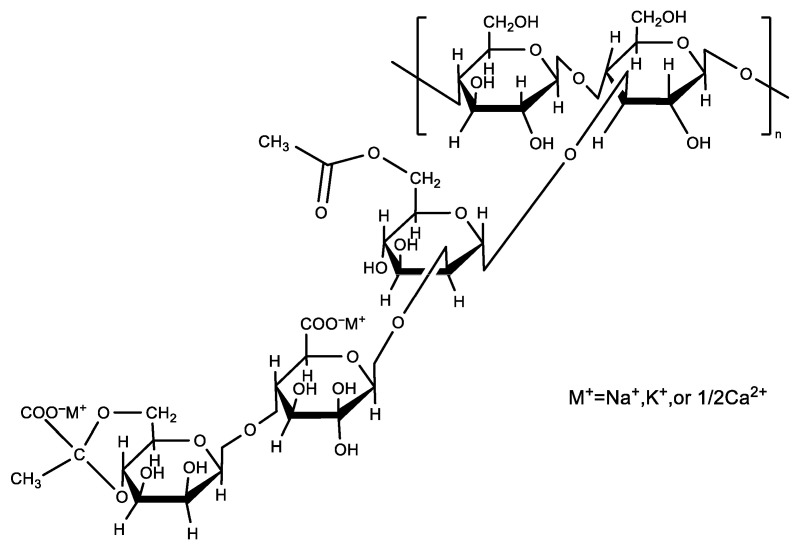
Molecular structure of XG.

For packaging applications, the integration of AgNPs into freestanding films is essential. Polyvinyl alcohol (PVA), stands out among polymer candidates due to its eco-friendly packaging [20]. It has attracted growing interest in the field of antimicrobial food packaging [21,22,23,24]. Owing to its excellent film-forming ability, biocompatibility, and biodegradability, PVA has been widely applied in food packaging, biomedical, and construction materials [25,26,27]. Additionally, montmorillonite (MMT) has shown great potential in enhancing the functional properties of PVA-based films [28]. PVA is often blended with materials such as starch and chitosan [29] to improve its mechanical performance [30]. Blending PVA with xanthan gum (XG) can also produce enhanced composite films. The experimental results of Chen et al. [31] reveal that PVA/XG composite films have superior character in food packaging, storage, and biodegradability. XG is a microbial polysaccharide derived from the fermentation of sugars by *Xanthomonas* spp. The main chain of XG comprises D-glucose units linked by *β*-1, 4-glycosidic bonds as shown in Figure 1. It shows an exceptional compatibility, solubility, and stability under conditions of low concentrations. It is non-toxic, non-allergenic, and widely utilized as an additive in diverse industrial and biomedical applications such as food packaging, cosmetics, water-based coatings, toiletries, and drug delivery [32].

In this study, *Myrica rubra leaf extract* (*MRLE*), an agricultural byproduct, was employed for the first time as a reducing agent for the green synthesis of AgNPs. The novel antimicrobial films were fabricated using solution casting by simultaneously incorporating *MRLE*-synthesized AgNPs and MMT into a PVA/XG matrix. Key parameters affecting AgNPs synthesis, including reaction time, temperature, and AgNO_3_ concentration, were systematically investigated. The control and composite films were comprehensively characterized for physicochemical properties, antimicrobial activity, and fruit preservation performance, highlighting their potential for application in advanced food packaging.

## 2. Materials and Methods

### 2.1. Materials

PVA (AR) is from Tianjin Damao Chemical Reagent Factory (Tianjin, China). and XG (food grade) is from Henan Wanbang Industry Co., Ltd. (Shangqiu, China). MMT K-10 (surface area 240 m^2^/g) is produced by Shanghai Aladdin Reagent Co., Ltd. (Shanghai, China). AgNO_3_ (AR) comes from Shenzhen Bolinda Technology Co., Ltd. (Shenzhen, China). Glycerol (AR) was obtained Tianjin Damao Chemical Reagent Factory. Nongfu Spring purified water is made by China Resources Nongfu Spring Beverage Co., Ltd. (Hangzhou, China). 2,6-Dichloroindophenol is from Yuan Ye Biotechnology Co., Ltd. (Shanghai, China). Oxalic acid dehydrate is produced by China National Pharmaceutical Group Chemical Reagent Co., Ltd. (Shanghai, China). Sodium hydroxide is provided by Tianjin Bodhi Chemical Co., Ltd. (Tianjin, China). Ascorbic acid is supplied by Xilong Scientific Chemicals (Yulin, China).

### 2.2. Preparation and Characterization of Plant Extract

**Figure 2:**
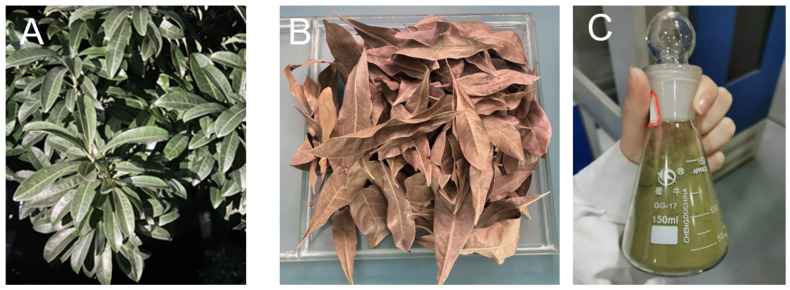
(**A**) Fresh *Myrica rubra* leaves; (**B**) Dried *Myrica rubra* leaves; (**C**) *Myrica rubra* leaves powder.

*Myrica rubra leaves* were collected from the campus of Hunan University of Technology under controlled conditions and washed with distilled water to clear away dust and visible particles. Subsequently, the leaves were dried under no-light conditions, then ground into a refined powder, and stored in an airtight container. Taking 100 g plant powder to mix with 300 mL 60% ethanol, we heated them at 60 °C for 1.5 h and then implemented a filtering and centrifuging process. Finally, the plant extract was stored at 4 °C. Figure 2 shows the different states of *Myrica rubra leaves* during the process.

A QTOF X500B mass spectrometer (SCIEX, Framingham, MA, USA) with an Exion AD UPLC system was used for sample analysis. Separation was performed by using a HSS T3 column (2.1 × 100 mm, 1.8 µm) (Waters, Milford, MA, USA) with a mobile phase of water (0.05% formic acid) and acetonitrile (0.05% formic acid), under a gradient elution (5–98%) at a speed of 0.3 mL/min for over 42 min and a temperature of 40 °C. The injection sample volume was 2 µL, and the autosampler was maintained at 4 °C. The QTOF X500B, equipped with an ESI source in a positive ion mode, was operated at a spray voltage of 5500 V, in which curtain gas was 35 psi, both GS1 and GS2 were 50 psi, and the ion source temperature was 500 °C. Compound characterization was performed using SCIEX OS 1.4 software, referring to databases such as Metlin, MassBank, and SciFinder Scholar.

### 2.3. Synthesis and Characterization of AgNPs

**Figure 3:**
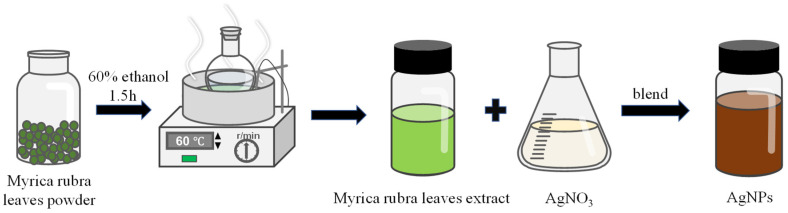
Synthesis process of AgNPs.

Modified AgNPs were synthesized based on the method reported by Alomar and Kora [33,34]. During the modified procedure, *MRLE* was mixed with AgNO_3_, following a rule of volume ratio of 1:9. The solution was placed in a water bath with continuous stirring until the color of solution changed to reddish-brown. Then, the prepared reddish-brown AgNP solution was centrifuged at 8000 rpm for 15 min and freeze-dried. The synthesis process is illustrated in Figure 3.

Optimization of the parameters was achieved using response surface methodology. A three-level, three-variable Box–Behnken factorial design (BBD) was selected to determine the best combination of AgNP size. AgNO_3_ concentration (A), temperature (B), and time (C) were selected as independent variables. The range and central point of the three independent variables presented in Table 1 are based on references and results of our preliminary single-factor experiments. Absorbance was selected as the response (Y).

#### 2.3.1. Structural Characterization of AgNPs

The microscopic morphology of AgNPs was observed using a transmission electron microscope (TEM, JEOL 2011, JEOL Ltd., Tokyo, Japan) operated at 200 kV, with a point resolution of 0.23 nm and coefficient of 1.0 mm. The structure of AgNPs was analyzed using Fourier transform infrared spectrometry (FTIR, Nicolet iS10, Thermo Fisher Scientific Inc., Waltham, MA, USA), while the change in crystal structure was assessed using X-ray diffraction (XRD, Thermo K-Alpha, Thermo Fisher Scientific, Inc.)

#### 2.3.2. Antimicrobial Activity of AgNPs

The antimicrobial activity of AgNPs synthesized using *MRLE* was evaluated against *Escherichia coli* (*E. coli*) and *Staphylococcus aureus* (*S. aureus*) with the paper disk diffusion method [35]. Pure microbial cultures were subcultured on nutrient agar at 37 °C [36]. Sterile cotton swab was used to evenly spread each strain on separate substrates. The substrates were incubated at 37 °C for 72 h to ensure maximum growth of bacterial colonies. Subsequently, 10 μL of film solution at different concentrations was applied to the filter paper discs. The plates were incubated upside down for 24 h, and the inhibition zone diameters were measured. Statistical differences between groups were analyzed using analysis of variance (ANOVA) in SPSS 24.0, with significance set as *p* < 0.05.

### 2.4. Preparation of Composite Films

Following the experimental protocol of Chen et al. [31], 1 g XG was dispersed in 200 mL distilled water with magnetic stirring (DF-101S, Hangzhou Jer Experimental Equipment Co., Ltd., Hangzhou, China) at a speed of 500 rpm. Simultaneously, 15 g PVA particles were dissolved in 500 mL distilled water and stirred at 500 rpm using a magnetic stirrer at 95 °C for 2 h. PVA and XG solutions were mixed at a dry matter ratio of 3:1. Glycerol (equivalent to 8% of PVA and XG) was adopted as a plasticizer. The PX film-forming solution was then thoroughly mixed and left to degas. The solution was cast evenly onto a glass substrate and dried in an oven (DHG-9420B, Shanghai Peiyin Experimental Instrument Co., Ltd., Shanghai, China) at 50 °C for 24 h. The dried film was peeled off and stored in sealed containers for subsequent analysis.

Briefly, 3 g MMT was dispersed in 100 mL deionized water and stirred for 8 h. The dispersed MMT was reacted with a 2 mM AgNO_3_ solution and continuously stirred at 70 °C for 8 h to form a AgNO_3_@MMT slurry. As a comparison, the dispersed MMT was reacted with a AgNP solution and continuously stirred at 70 °C for 8 h to obtain a AgNPs@MMT slurry. Then, different types and amounts of components were added to the PX film-forming solution to obtain a series of composite films. Table S1 shows the abbreviations and ratios of components for various composite films.

### 2.5. Characterization of Composite Films

The morphology, water solubility, mechanical, and barrier properties of the films were determined, and these steps are detailed in Supporting Information S1.

#### 2.5.1. Antimicrobial Properties

The antimicrobial efficacy of the film solutions was assessed using the filter paper diffusion method. For detailed operational procedures, please refer to Section 2.3.2. The key differences are reflected in the sample cases.

#### 2.5.2. Specific Migration Experiment

A 10% ethanol solution was used as the food simulant. PA2, PAM2, and PANM2 films were cut into 50 × 60 mm^2^ rectangles and immersed in 50 mL of 10% ethanol. Each film type was tested in triplicate to ensure reproducibility. The vessels were sealed with the corresponding films and incubated at 25 °C in a constant temperature chamber for 10 days. Silver migration from the films was analyzed using inductively coupled plasma mass spectrometry (ICP-MS, iCAP 7400, Thermo Fisher Scientific).

#### 2.5.3. Molecular Dynamics Simulation

As shown in Figure S1, to investigate diffusion mechanism at the molecular level, amorphous cell models of PVA/XG-AgNPs, PVA/XG-AgNPs@MMT, and PVA/XG-AgNO_3_@MMT with periodic boundary conditions were constructed by molecular dynamics simulation (MD). After performing a geometric optimization procedure, the model underwent annealing treatment. MD simulations were used to analyze the diffusion behavior of AgNPs within the different composite matrices. Interaction energy between Ag particles and substrates or MMT, motion trajectories, and free volume fractions of the cell models were calculated.

### 2.6. Application in Grape and Banana Preservation

**Figure 4:**
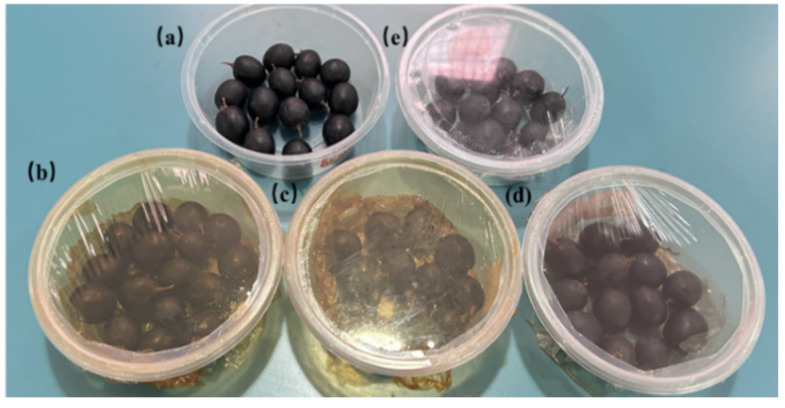
The schematic diagram of grape preservation packaging groups, which were covered by (**a**) blank; (**b**) PA film; (**c**) PAM film; (**d**) PANM film; and (**e**) P film.

Grapes of uniform maturity, size, and without visible damage were selected for testing. The grapes were divided into five groups, including the blank control, P, PA, PAM, and PANM groups, for preservation treatments. Each group of grapes was placed in a box lined with a bottom film and covered by the corresponding film as shown in Figure 4. All samples were stored and tested at room temperature for 8 days.

**Figure 5:**
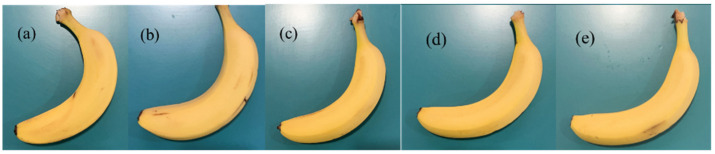
The schematic diagram of banana preservation packaging groups, which were covered by (**a**) blank; (**b**) PA film; (**c**) PAM film. (**d**) PANM film; and (**e**) P film.

Banana treatment followed the methodology of Rodrigues et al. [37]. The same selection criterion employed for grapes was also applied to banana experiment. In total. 75 bananas were divided into five groups, including the blank control (uncoated), P, PA, PAM, and PANM groups as shown in Figure 5. All tests were conducted in triplicate. Daily changes in appearance and physicochemical properties of coated and uncoated fruits were recorded for 5 days.

Weight loss, firmness, pH, vitamin C (Vc) content, and titratable acidity (TA) were measured, with detailed methods provided in Supporting Information S2.

SPSS software was used for statistical analysis. Data were presented as an expression of mean value ± standard deviation. The LSD method was employed for significant difference testing, where differences were considered significant at *p* < 0.05.

## 3. Results and Discussion

### 3.1. Analysis of Plant Extract

The *MRLE* was analyzed using UHPLC-QTOF. Table 2 and Figure S2 list a higher level of detected bioactive compounds, including phenolics and flavonoids such as myricitrin, rutin, and luteolin-7-glucoside. These compounds contribute significantly to the extract’s bioactivity and exhibit strong reducing and stabilizing capacities. Similar findings were reported by Ejaz [38].

**Figure 6:**
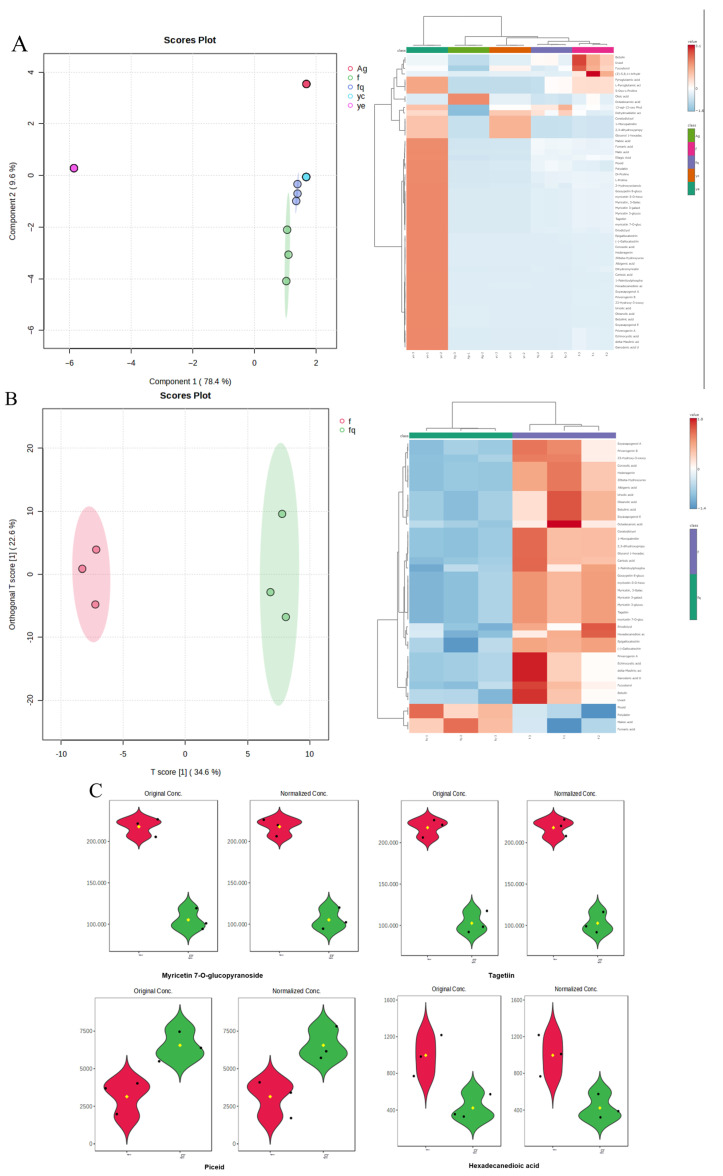
(**A**) Scatter plot of principal component analysis scores and heat map of the mixture before and after the reaction. (**B**) Scatter plot of principal component analysis scores and heat map of the PCA score scatter plot and heat map of the 60% ethanol, leaf extract, pre- and post-reaction mixture, and AgNPs. (**C**) Changes in the composition of certain compounds violin diagram. f indicates post-reaction, fq indicates pre-reaction, ye represents waxberry leaf extract, Ag denotes silver nitrate, and yc represents ethanol.

Figure 6A shows significant changes in carbohydrate and flavonoid content before and after the reaction. These changes may result from the compounds’ involvement in AgNP synthesis or partial adsorption onto the nanoparticles. Figure 6C further demonstrates that the concentrations of certain compounds, such as myricetin 7-O-glucopyranoside and tagetin, were significantly reduced, whereas the concentrations of compounds like piceid and hexadecanedioic acid showed a slight increase. However, Figure 6B indicates that the nanoparticle surface composition showed little correlation with the leaf extract components. This suggests that the component changes were not primarily due to adsorption onto AgNPs. Rather, the changes likely resulted from their chemical involvement in the nanoparticle formation process.

### 3.2. Synthesis of AgNPs

#### 3.2.1. Analysis of Box–Behnken Design

**Figure 7:**
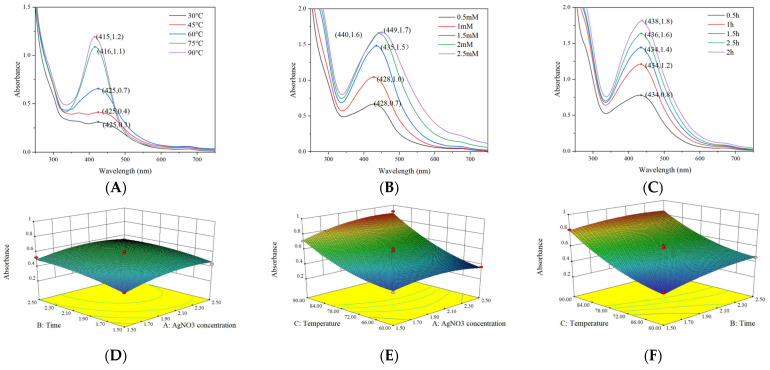
UV–Vis spectrum of AgNPs solution samples in varied reaction temperatures (**A**), diverse AgNO_3_ concentrations (**B**), and different reaction time (**C**). Response surface and contour of the interaction of AgNO_3_ concentration (**D**), reaction temperatures (**E**) and reaction time on particle size (**F**).

Ultraviolet–visible spectroscopy (250–750 nm) analysis (Figure 7A–C) revealed a distinct surface plasmon resonance absorption peak at 420 nm for the synthesized AgNPs, indicating excellent dispersion characteristics, which is consistent with previously reported features of plant extract-mediated AgNP synthesis [39]. As shown in Table S2, a three-factor BBD was employed to optimize the synthesis parameters, yielding the following quadratic regression model: Absorbance = 0.56 + 0.037A + 0.052B + 0.22C − 0.014AB + 0.043AC − 0.026BC − 0.062A^2^ − 0.037B^2^ + 0.079C^2^. Analysis of variance (Table S3) demonstrated the model’s high significance (F = 51.62, *p* < 0.0001) with non-significant lack of fit (*p* = 0.3434) and excellent goodness of fit (*R*^2^ = 98.52%), confirming its reliability for optimizing AgNPs synthesis parameters [40,41]. Figure 7D–F shows that the interaction between AgNO_3_ concentration and reaction temperature exhibits the most significant effect on particle size, whereas the interactions between AgNO_3_ concentration and reaction time as well as between reaction temperature and reaction time are not statistically significant.

#### 3.2.2. Microscopic Structure Analysis of AgNPs

**Figure 8:**
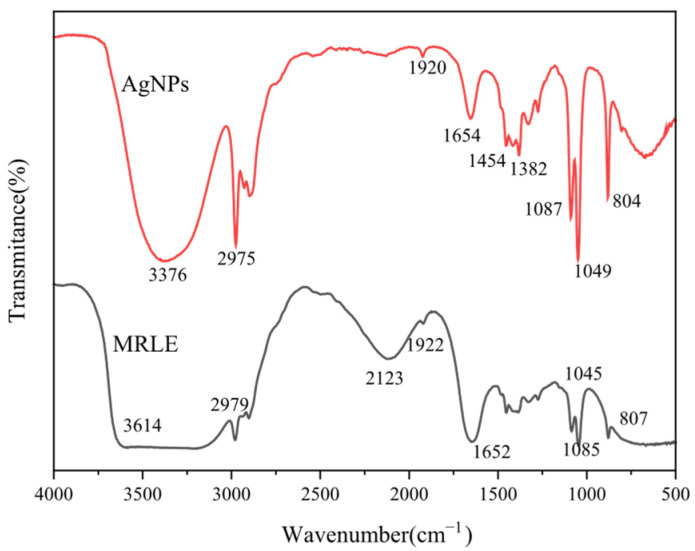
FTIR spectra of AgNPs and *MRLE*.

Figure 8 presents the FTIR spectra of *MRLE* and the biosynthesized AgNPs. In the *MRLE* spectrum, a broad absorption band at 3614 cm^−1^ is attributed to O–H stretching vibrations, indicating the presence of alcohols and phenolic compounds. After nanoparticle synthesis, this band shifts to 3376 cm^−1^ with a noticeable decrease in intensity, suggesting the involvement of hydroxyl groups—likely from phenolics—in the reduction and stabilization of AgNPs [42]. Both *MRLE* and AgNP spectra exhibit C–H stretching bands near 2979 cm^−1^ and 2975 cm^−1^, respectively, characteristic of aliphatic hydrocarbons. Peaks observed at 1045 cm^−1^ (*MRLE*) and 1049 cm^−1^ (AgNPs) correspond to C–O stretching vibrations, typically associated with alcohols, ethers, or esters. The appearance of absorption bands around 1652–1654 cm^−1^ may be related to C=O stretching of amide I or conjugated carbonyl groups, which are often linked to proteins or aldehydes participating in the reduction and capping processes.

Additional peaks at 1382 cm^−1^ and 1454 cm^−1^ in the AgNP spectrum may correspond to symmetric and asymmetric bending vibrations of –CH_3_/–CH_2_ groups, possibly from residual plant metabolites. The band near 1087 cm^−1^ (AgNPs) may also reflect C–N stretching from amine groups, indicating that proteins or amino acids from the extract may contribute to nanoparticle stabilization through surface adsorption or capping interactions [43]. These results support the hypothesis that phenolic compounds, aldehydes, and amide-containing biomolecules play active roles in both the reduction of silver ions and the stabilization of AgNPs. However, detailed mechanistic studies are needed to fully understand these interactions.

**Figure 9:**
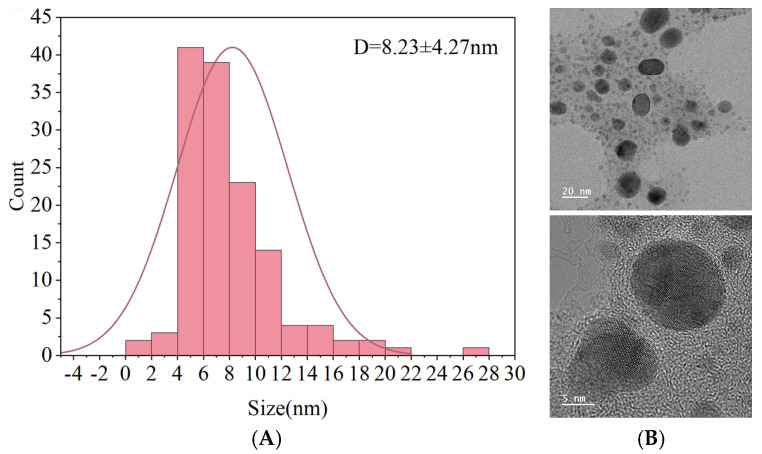
Particle size distribution (**A**) and TEM image (**B**) of AgNPs.

TEM images were analyzed using ImageJ 1.x software, and the particle size distribution was obtained by measuring over 150 individual nanoparticles. The average particle diameter was calculated to be 8.23 ± 4.27 nm, as shown in Figure 9A. These results are consistent with previous reports on green-synthesized AgNPs, which commonly exhibit spherical morphology in the range of 7.7–42.9 nm [44]. Figure 9B shows representative TEM images at different magnifications, with embedded scale bars of 20 nm and 5 nm, respectively, further confirming the spherical shape and nanoscale dimensions of the particles.

**Figure 10:**
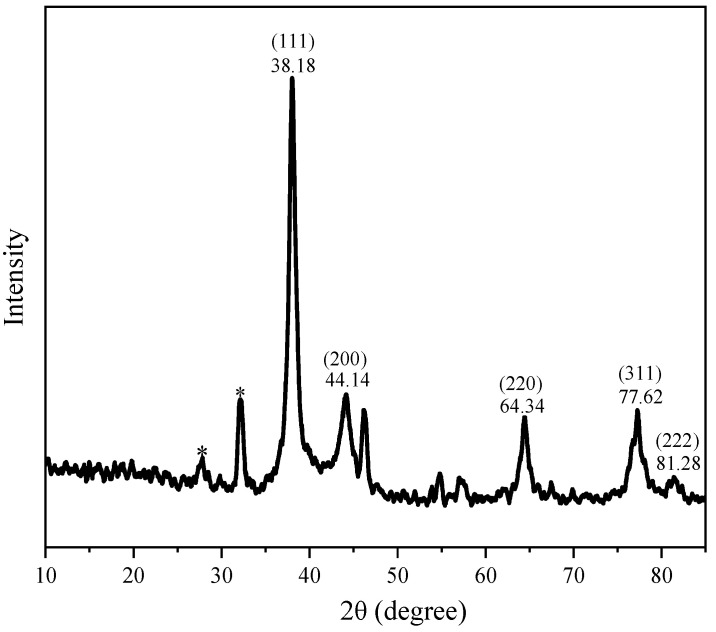
X-ray diffraction pattern of AgNPs.

Figure 10 plots XRD spectrum of AgNPs. The XRD spectrum displays characteristic diffraction peaks at 38.18°, 44.14°, 64.34°, 77.62°, and 81.28° in the 2θ region, which corresponds to the (111), (200), (220), (311), and (222) planes of silver, respectively. Thus, we can draw the conclusion that AgNPs are formed. The results are consistent of the reports of Joint Committee on Powder Diffraction Standards File No. 04-0783 for silver (face centric cubic) [45]. Except the characteristic Bragg peaks of AgNPs, additional peaks between 20° and 30° suggest the presence of reducing and capping moieties on the surface of the nanoparticles. These findings are in agreement with earlier reports on AgNPs synthesized using *Caesalpinia pulcherrima* flower extract [43] and *Moringa oleifera* flower [46].

#### 3.2.3. Antimicrobial Activity of AgNPs

**Figure 11:**
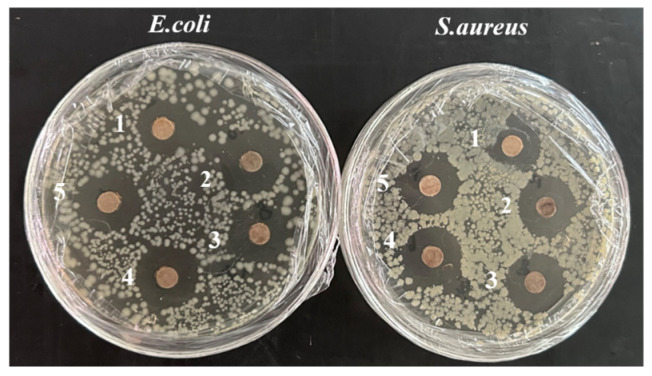
Zone of inhibition of AgNPs against *E. coli* and *S. aureus*.

Table 3 shows the antimicrobial activity of AgNPs. *S. aureus* showed the highest sensitivity (20.21 mm), followed by *E. coli* (18.79 mm), as illustrated in Figure 11. Increasing AgNPs concentration enhanced inhibition against both Gram-negative *E. coli* and Gram-positive *S. aureus*, consistent with findings reported by Algarni [47]. The antimicrobial effect of AgNPs primarily results from the electrostatic interaction with the bacterial surface. The nanoscale of AgNPs prompts their penetration to bacterial cell wall, disrupting DNA aggregation and replication. Therefore, AgNPs possess the ability of inhibiting microbial growth and leading to cell death [48,49].

### 3.3. Measurement of Physical and Chemical Indexes of Composite Film

#### 3.3.1. Structural Characterization of Composite Films

**Figure 12:**
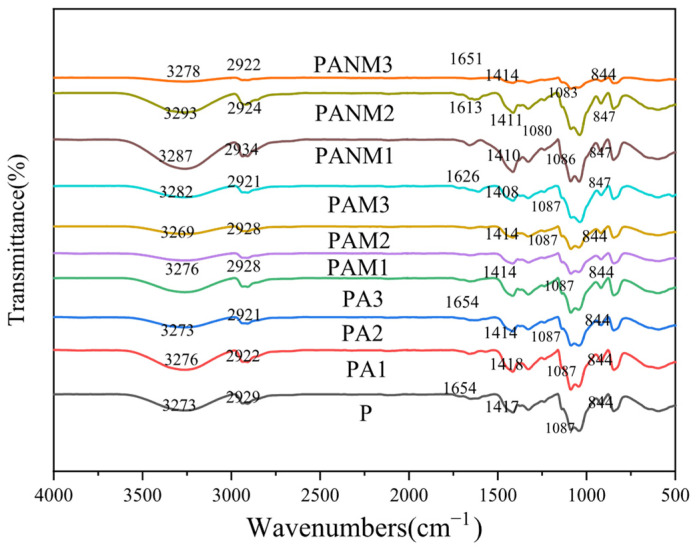
FTIR spectra of pure PVA, XG, and composite films.

The ATR-FTIR spectra of composite films are shown in Figure 12. The characteristic peak of the –OH stretching vibration for P film at 3273 cm^−1^ was observed. The vibration band at 2929 cm^−1^ was attributed to the asymmetric stretching of C–H in alkyl groups. Peaks at 1417 cm^−1^ and 1087 cm^−1^ correspond to the stretching of C–H and C–O–C groups. The minor peak at 844 cm^−1^ corresponds to the C–C main chain of PVA. Compared to the P film, the nanocomposite films showed no new peaks, suggesting no chemical bond formation—similar to findings reported by Tran Pham et al. [24] for PVA/D–glucose/gelatin–AgNP composites. They suggested that there were no chemical interactions between PVA and AgNPs. A slight shift in the –OH peak in PAM and PANM films with 3% MMT may indicate interactions between PVA and MMT.

In PAM films, the 2929 cm^−1^ peak shifted to 2922 cm^−1^. The –OH stretching peak also shifted from 3273 to 3293 cm^−1^ in PAM and PANM films. This shift may result from AgNPs altering hydrogen bonding among PVA chains.

**Figure 13:**
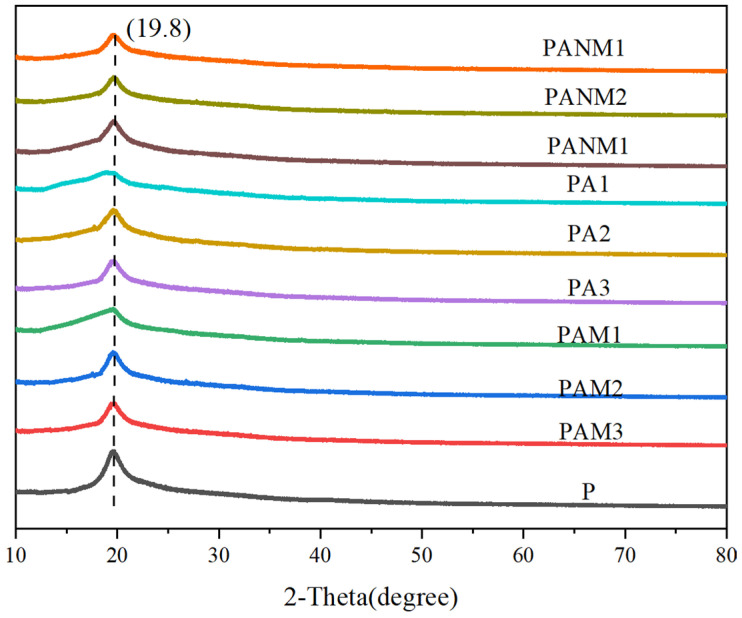
X-ray diffraction patterns for composite films.

XRD patterns of the nanocomposite films are shown in Figure 13. A sharp peak at 2θ of 19.4° for the P film indicates the crystallinity of PVA. As shown in Figure 13, AgNPs, MMT, and AgNO_3_ did not shift the peak position, indicating minimal impact on polymer crystallinity. This suggests that nanofillers affect macroscopic properties through physical interaction rather than altering the crystal structure. Alternatively, the low concentration of nanofillers do not induce a detectable change in the XRD pattern. Similar trends have been observed in other composite films [50].

**Figure 14:**
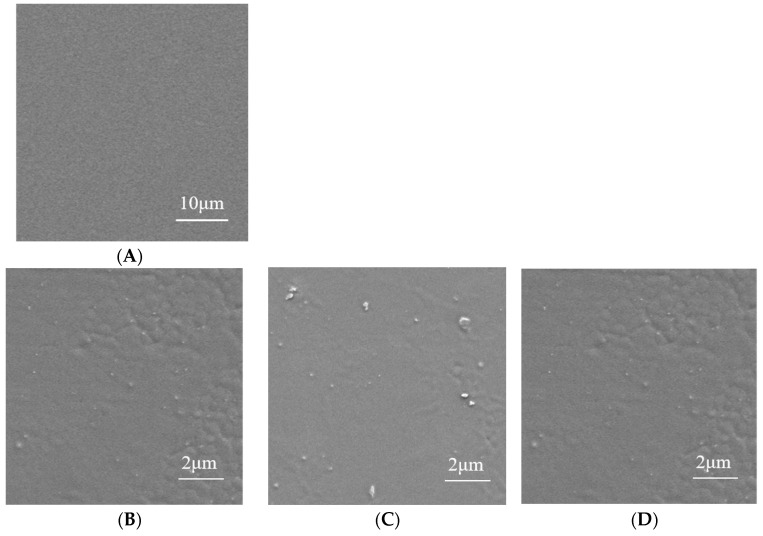

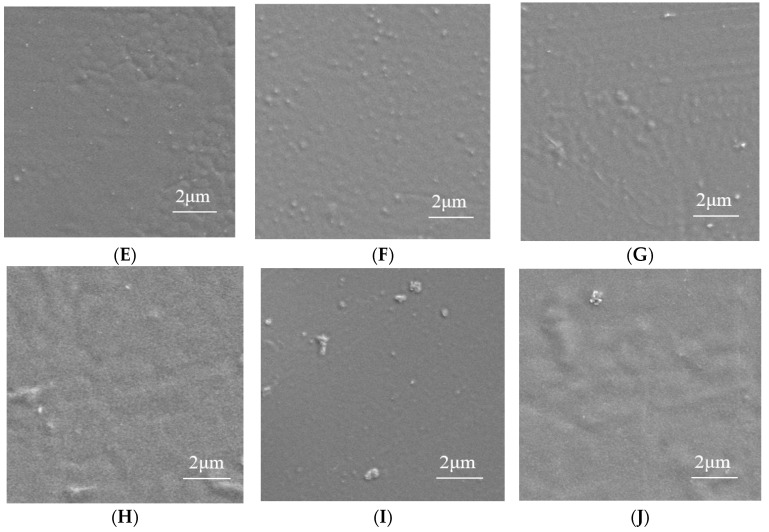
SEM images of surface of P film (**A**) and composite films PA1 (**B**), PA2 (**C**), PA3 (**D**), PAM1 (**E**), PAM2 (**F**), PAM3 (**G**), PAMN1 (**H**), PANM2 (**I**), and PAMN3 (**J**).

SEM images provide microscopic structural information regarding the compatibility between film-forming substrates. SEM images of the composite films are shown in Figure 14. For the P film, the smooth, dense, and uniform surface without irregularities indicates good compatibility between PVA and XG. In contrast, the addition of AgNPs in the PA film leads to a rough and uneven surface. Similar surface features were reported in PVA films containing ginger extract, AgNPs, and MMT [51].

#### 3.3.2. Mechanical Properties

**Figure 15:**
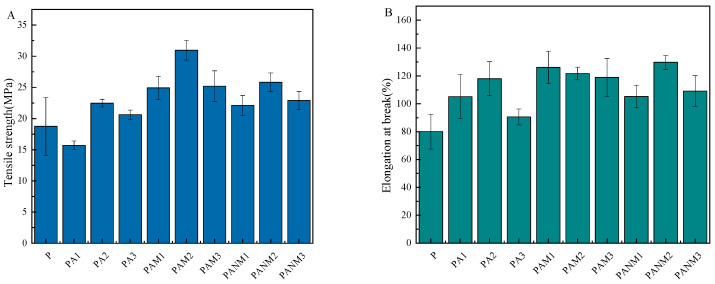
The tensile strength of the film (**A**); the elongation at break of the film (**B**).

The mechanical strength of composite films is shown in Figure 15. The addition of AgNPs and MMT yields a significant increase in the mechanical strength of PA, PAM, and PANM films to varying degrees. As compared with the P film, the introduction of AgNPs in PX lead to an increase in tensile strength (TS) and elongation at break (EAB), particularly in PAM2, for which TS and EAB increase by 65.19% and 52.10%, respectively. PANM2 film exhibits a 37.70% increase in TS (*p* > 0.05). The enhancement in TS and EAB is attributed to interactions between PVA matrix and AgNPs as well as MMT. Similar results were found when AgNPs, MMT, and ginger extract were incorporated into PVA films [51].

#### 3.3.3. Optical Property

**Figure 16:**
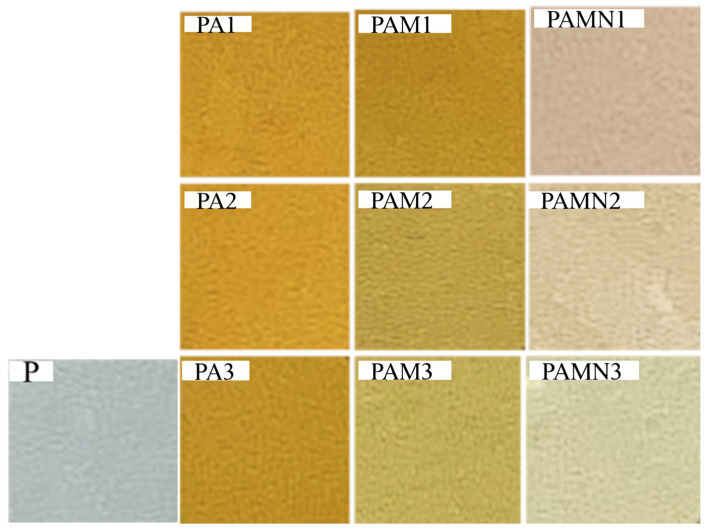
Image of composite films.

Figure 16 displays images of composite films with varying concentrations of AgNPs, while Table 4 lists detailed parameters for these composite films. The P film exhibits excellent transparency, whereas PA and PAM films are brownish-yellow, reflecting a decrease in L* value and an increase in a* and b* values. A similar phenomenon has been reported in the experiment involving the incorporation of AgNPs into composite films based on PVA/thermoplastic starch [52].

As shown in Table 4, the transmittance of composite films at 280 nm and 600 nm represents the UV-blocking and transparency properties of the films. The addition of AgNPs leads to a decreasing effect on the transmittance of the composite films. The transparency of the P film is 90.36%, and that of PA film derived from the mixing of P film with AgNPs decreases significantly with increasing AgNPs concentration (*p* < 0.05).

The addition of 3% MMT in PAM further reduces the visible light transmittance to 70.64%, while that of MMT and AgNPs yields a significant decrease in transparency, 68.41% (*p* < 0.05), both of which confirm the superior light-blocking performance of the composite films. This light-blocking capability helps to reduce UV-driven reactions such as lipid peroxidation and discoloration; thus, these films exhibit promise in the application of food preservation [53].

#### 3.3.4. Barrier Performance

AgNPs and MMT are helpful to increase the diffusion path and tortuosity of water molecules in the film, thus the WVP of composite films is reduced to 52.81 g/(m^2^·24 h) as listed in Table 5. AgNPs can also fill the gaps inherently occurred in PVA/XG films. This can prolong the diffusion path of H_2_O molecules and hinder their permeation, which thus leads to lower WVP as compared with other films [51].

### 3.4. Moisture Absorption

Moisture absorption is a key factor of water resistance for food packaging films. Data listed in Table 6 show that PA and PAM films exhibit lower water absorption rates compared with P film. The moisture content of P film is 6.47%, and the incorporation of MMT into PA film leads to a much smaller water absorption rate of 4.10%. This is attributed to the formed tortuous paths when bypassing AgNPs and MMT, which significantly prolongs the diffusion path length. The previous report has shown a similar observation in polyethylene-based nanocomposite films having nanoclay and AgNPs [54]. Table 6 also demonstrates a decreasing swelling capacity of Ag@MMT composite films, likely due to the presence of Ag and MMT, which created convoluted paths for water molecules.

### 3.5. Antimicrobial Activity

**Figure 17:**
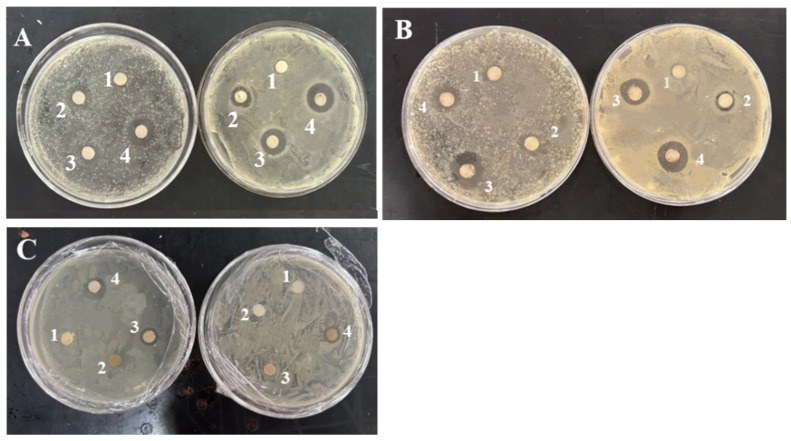
Inhibition of *E. coli* and *S. aureus* with membrane solution: (**A**) PA membrane solution (1 is P membrane solution control, 2 is PA3 membrane solution, 3 is PA2 membrane solution, 4 is PA1 membrane solution); (**B**) PAM membrane solution; (**C**) PANM membrane solution.

The antimicrobial activity of *E. coli* and *S. aureus* is evaluated for PA, PAM, and PANM membrane fluid compared with the control P. Clear inhibition zones are observed for PA, PAM, and PANM membrane fluid as depicted in Figure 17. It is evidently observed from Figure 17 that increasing concentrations of AgNPs leads to significantly larger inhibition zone diameters. The nanoscale size of AgNPs enables them to penetrate bacterial cell walls, where they can interfere with DNA aggregation and replication processes. As a result, AgNPs exhibit strong antimicrobial activity by inhibiting microbial growth and ultimately inducing cell death [48,49]. Among these, PA2 composite film exhibited the largest inhibition zones of 18.56 mm (*E. coli*) and 20.73 mm (*S. aureus*). As the concentration of Ag@MMT in the PVA film increases, the inhibition zone also becomes larger. This positive antimicrobial effect has also been reported by Yahia et al. [52] for PVA/modified starch/AgNPs composite films, having a good antimicrobial activity against *E. coli* (10 mm), *S. aureus* (7 mm), and *Candida albicans* (27 mm). AgNPs and MMT play complementary roles in enhancing film performance. AgNPs exhibit antimicrobial activity by disrupting bacterial membranes, while MMT improves their dispersion and stability through interlayer confinement. The layered structure of MMT also increases diffusion path tortuosity, enhancing barrier and mechanical properties. Although their combination shows promising synergistic effects, further research is needed to optimize synthesis conditions and ensure consistent functionality across applications.

### 3.6. Specific Migration Experiment

Specific migration is a crucial element in food safety assessment. Table 7 lists the specific migration limits of Ag particles from PA, PAM, and PANM composite films when using a 10% ethanol food simulant at 25 °C for 10 days. The results demonstrate that the migration amount of Ag particles from all membranes is far below the safety limit proposed by the European Food Safety Authority (10 mg/kg), thus the films are adequately qualified to be used as food contact materials. From the table, it is found that Ag particles show the highest migration in PA2. Ag particles in PAM2 exhibit a lower migration than PA2 due to the extra MMT. Ag particles in PANM show the lowest migration, possibly due to adsorption by MMT and ion exchange interactions.

### 3.7. MD Results

**Figure 18:**
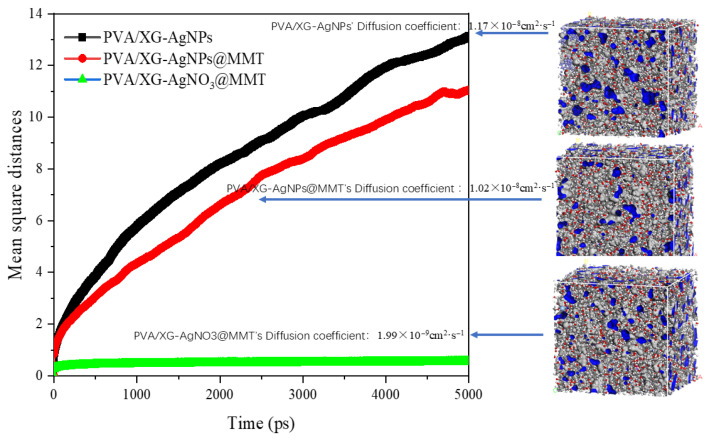
Free volume in PVA/XG-AgNPs, PVA/XG-AgNPs@MMT, and PVA/XG-AgNO_3_@MMT for Ag clusters.

MD simulations are carried out to find out a micro-level explanation for the migration phenomenon. For simulation details, please refer to Supporting Information S2. The simulation results show that Ag particles exhibit the highest diffusion coefficient in the PVA/XG-AgNPs crystalline cell, while the diffusion coefficient decreases in cells when adding MMT. It suggests that MMT has an inhibitory effect on AgNPs diffusion. Figure S3 exhibited the trajectories of Ag particles in the crystal cell at 298 K in different modules. The free volume fractions of PVA/XG-AgNPs, PVA/XG-AgNPs@MMT, and PVA/XG-AgNO_3_@MMT are calculated to be 13.52 ± 0.37, 13.10 ± 0.76, and 12.99 ± 0.81, respectively. This indicates that the involvement of MMT can reduce the free volume fraction of the cells and thus lower the diffusion capability of AgNPs as illustrated in Figure 18.

The non-bonding interactions between Ag particles and the matrix are depicted in Table 8. The results indicate that MMT exhibits negligible interactions with Ag atoms. However, the addition of MMT enhances the interaction energy between Ag atoms and PVA/XG, with an amplitude of 16.85%. This means that MMT has an effect on the interaction between Ag atoms and PVA/XG to prevent Ag atoms from diffusing. In the PVA/XG-AgNO_3_@MMT structure, the electrostatic interaction between Ag^+^ and MMT significantly increases, which tightens the binding of Ag^+^ in the polymer matrix. Thus, the diffusion coefficient of Ag^+^ is greatly reduced as characterized in the experiment.

### 3.8. Freshness Performance

**Figure 19:**
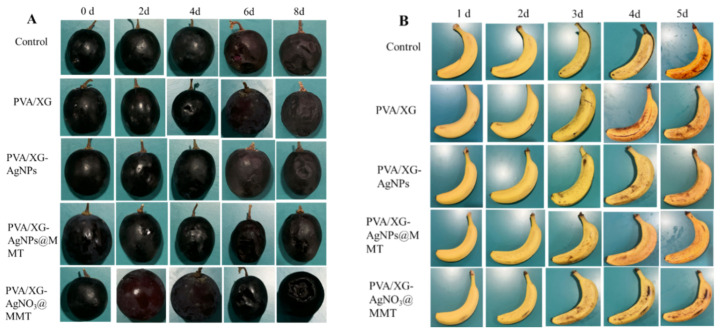
Changes in appearance of grape samples (**A**) and banana samples (**B**).

**Figure 20:**
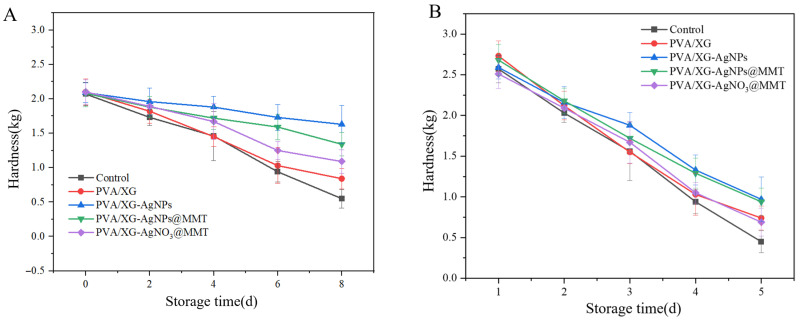
The hardness of (**A**) grapes and (**B**) bananas samples.

**Figure 21:**
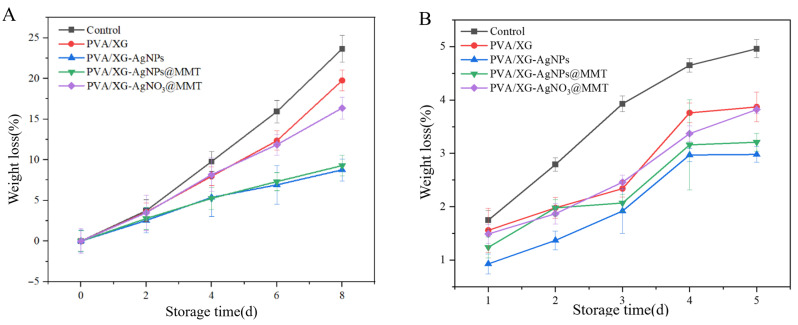
The weight loss of (**A**) grapes and (**B**) bananas samples.

As shown in Figure 19, grapes and bananas exhibited differential deterioration after 8 and 5 days of storage across packaging films or coating, primarily manifested as microbial-induced decay and mold spots. PA and PAM groups significantly preserved visual quality through physical barrier effects and AgNPs’ antimicrobial activity. Hardness analysis (Figure 20) demonstrated PA films most effectively maintained grape firmness (0.46 kg/cm^2^ reduction), while both PA and PAM groups delayed banana softening by modulating gas exchange. Weight loss (Figure 21) was minimized by PAM films through moisture retention, consistent with previous nanocomposite studies [55]. Quality parameters including titratable acidity (Figure S4), TSS (Figure S5), and Vc content (Figure S6) were better preserved in P and PAM groups. Notably, PA-coated bananas showed minimal TSS increases. pH monitoring (Figure S7) revealed that AgNPs slowed grape acidification, while all treatments similarly moderated banana pH elevation. These results collectively demonstrate PA and PAM films’ superior preservation efficacy through microbial inhibition and metabolic regulation.

## 4. Conclusions

In this study, AgNPs were green-synthesized using a 60% ethanol extract of *Myrica rubra* leaves, and a series of novel antibacterial composite films based on PVA and XG were developed using the solution casting method. UHPLC-QTOF analysis revealed the extract’s abundance of phenolic and flavonoid compounds, which played a positive role in the synthesis of AgNPs. PVA/XG films exhibited excellent film-forming properties, and the addition of AgNPs and MMT contributed to the improvement of antimicrobial and mechanical properties, demonstrating potential for food packaging optimization. FTIR analysis confirmed interactions among PVA, XG, AgNPs, and MMT, whereas XRD and SEM analyses demonstrated the uniform distribution of nano-fillers within the polymer matrix. Antimicrobial tests revealed significant bactericidal effects against *E. coli* and *S. aureus*. Specific migration tests revealed varying levels of AgNP migration in different composite films, all within the permissible limits for food contact materials. The migration behavior of Ag was further analyzed using MD simulation. Furthermore, the application of nano-composite films and coatings to grape and banana preservation demonstrated their ability to effectively delay ripening and decay, confirming the potential of PVA/XG-AgNPs@MMT films for functional food packaging. Future research should systematically evaluate AgNP migration kinetics under different temperature and humidity conditions to ensure long-term food safety.

## Figures and Tables

**Table 1:** Factors and levels of response surface analysis.

Independent Variables	Low Level	Level
AgNO_3_ concentration (mMol/L)	1.5	2
Time (h)	1.5	2
Temperature (°C)	60	75

**Table 2:** Partial composition of *MRLE*.

NO.	Name	Retention Time	Area
1	Myricitrin	9.96	3.49 × 10^7^
2	Isoorientin	11.56	1.40 × 10^7^
3	Astragalin	11.56	1.40 × 10^7^
4	(-)-Epigallocatechin Gallate	7.90	1.01 × 10^7^
5	Gallic acid	2.43	9.32 × 10^6^
6	Carissic acid	39.08	8.79 × 10^6^
7	Quinic acid	0.88	5.19 × 10^6^
8	Asiatic acid	30.28	4.64 × 10^6^
9	Monooctyl phthalate	35.63	3.20 × 10^6^
10	Hederagenin	34.94	2.81 × 10^6^
11	Corosolic acid	34.94	2.81 × 10^6^
12	20beta-Hydroxyursolic acid	34.94	2.81 × 10^6^
13	Albigenic acid	34.94	2.81 × 10^6^
14	23-Hydroxy-3-oxocycloart-24-en-26-oic acid	34.94	2.80 × 10^6^
15	Quercitrin	11.63	2.29 × 10^6^

**Table 3:** Antimicrobial activity of AgNPs.

System	Concentration of AgNPs (mMol/L)	*E. coli* (mm)	*S. aureus* (mm)
1	0.5	15.76	17.42
2	2	18.65	18.82
3	4	18.68	18.84
4	6	17.17	20.21
5	8	18.79	18.57

**Table 4:** Color and transmittance of composite films.

Samples	L*	a*	b*	T280 (%)	T600 (%)
P	92.04 ± 0.28 ^b^	−1.55 ± 0.24 ^b^	−4.36 ± 0.46 ^b^	59.45 ± 0.87 ^b^	90.36 ± 0.64 ^a^
PA1	68.91 ± 0.79 ^a^	18.21 ± 0.41 ^a^	37.91 ± 0.39 ^a^	10.34 ± 0.36 ^a^	75.67 ± 0.36 ^a^
PA2	65.62 ± 0.91 ^a^	19.38 ± 0.48 ^b^	39.07 ± 0.76 ^b^	9.97 ± 0.94 ^b^	71.49 ± 0.28 ^b^
PA3	61.18 ± 0.27 ^c^	23.17 ± 0.11 ^a^	37.36 ± 0.47 ^c^	8.56 ± 0.27 ^c^	68.41 ± 0.47 ^a^
PAM1	74.63 ± 0.39 ^a^	11.40 ± 0.18 ^a^	30.80 ± 0.26 ^a^	13.74 ± 0.66 ^a^	78.14 ± 0.33 ^c^
PAM2	70.11 ± 0.64 ^b^	13.57 ± 0.36 ^c^	31.91 ± 0.51 ^b^	11.61 ± 0.21 ^b^	74.83 ± 0.74 ^a^
PAM3	56.32 ± 0.67 ^a^	25.24 ± 0.77 ^b^	33.47 ± 0.56 ^c^	10.27 ± 0.46 ^c^	70.64 ± 0.58 ^b^
PANM1	82.06 ± 0.82 ^a^	3.86 ± 0.29 ^a^	2.93 ± 0.22 ^b^	29.47 ± 0.55 ^d^	85.76 ± 0.24 ^a^
PANM2	82.06 ± 0.82 ^a^	3.86 ± 0.29 ^a^	2.93 ± 0.22 ^b^	29.47 ± 0.55 ^d^	85.76 ± 0.24 ^a^
PANM3	82.06 ± 0.82 ^a^	3.86 ± 0.29 ^a^	2.93 ± 0.22 ^b^	29.47 ± 0.55 ^d^	85.76 ± 0.24 ^a^

^a–c^ indicate significant differences (*p* < 0.05) within column L*. ^a–c^ indicate significant differences (*p* < 0.05) within column a*. ^a–c^ indicate significant differences (*p* < 0.05) within column b*. ^a–d^ indicate significant differences (*p* < 0.05) within column T280. ^a–c^ indicate significant differences (*p* < 0.05) within column T600.

**Table 5:** Water vapor permeability of composite films.

Number	WVP (g/(m^2^·24 h))
P	62.68 ± 2.59 ^a^
PA1	60.80 ± 1.82 ^a,b^
PA2	60.29 ± 1.97 ^a,b^
PA3	60.30 ± 1.91 ^a,b^
PAM1	52.81 ± 2.31 ^c^
PAM2	55.00 ± 3.32 ^b,c^
PAM3	55.58 ± 6.04 ^a,b,c^
PANM1	60.85 ± 1.70 ^a,b^
PANM2	61.97 ± 4.63 ^a,b^
PANM3	60.85 ± 3.77 ^a,b^

^a–c^ indicate significant differences (*p* < 0.05).

**Table 6:** Water content and swelling of composite films.

Number	Water Content/%	Dissolution Rate/%
P	6.47 ± 0.93 ^a^	150.83 ± 0.42 ^a^
PA1	4.30 ± 0.73 ^b^	147.60 ± 0.29 ^b^
PA2	4.34 ± 0.55 ^b^	145.10 ± 0.34 ^b,c^
PA3	4.10 ± 0.47 ^b^	143.70 ± 0.50 ^b,c,d^
PAM1	4.13 ± 0.24 ^b^	138.40 ± 0.25 ^d,e,f^
PAM2	4.23 ± 0.44 ^b^	136.90 ± 0.21 ^e,f^
PAM3	4.59 ± 1.02 ^b^	132.80 ± 0.52 ^f^
PANM1	4.26 ± 0.48 ^b^	141.90 ± 0.22 ^b,c,d,e^
PANM2	4.24 ± 0.53 ^b^	142.40 ± 0.31 ^b,c,d,e^
PANM3	4.45 ± 0.36 ^b^	140.70 ± 0.08 ^c,d,e^

^a–b^ indicate significant differences (*p* < 0.05) within column water content. ^a–f^ indicate significant differences (*p* < 0.05) within column dissolution rate.

**Table 7:** Specific migration limits of AgNPs from PA, PAM, and PANM composites to food simulants at 25 °C for 10 d.

Number	Specific Migration (mg/kg)
PA2	0.25 ± 0.03
PAM2	0.19 ± 0.04
PANM2	0.074 ± 0.29

**Table 8:** Interface interaction energy of AgNPs in PVA/XG-AgNPs, PVA/XG-AgNPs@MMT, and PVA/XG-AgNO_3_@MMT.

Cell	Subject (A/B)	Eint (kcal/mol)	Elec in Eint (kcal/mol)	vdW in Eint (kcal/mol)
PA	Ag/polymer	−111.80	0.0000	−108.48
PAM	Ag/polymer	−134.47	0.0000	−131.37
PAM	Ag/MMT	−1.61	0.0000	−1.48
PAM	Ag/(polymer + MMT)	−133.73	0.0000	−130.42
PANM	Ag^+^/polymer	−604.01	−525.02	−75.94
PANM	Ag^+^/MMT	−791.15	−790.86	−0.12
PANM	Ag^+^/(polymer + MMT)	−1498.56	−1415.96	−79.30

## Data Availability

The original contributions presented in the study are included in the article/Supplementary Material, further inquiries can be directed to the corresponding author.

## Supplementary Materials

The following supporting information can be downloaded at: https://www.mdpi.com/article/10.3390/foods14142509/s1, Figure S1: PVA/XG-AgNPs model: (a) PVA/XG-AgNPs@MMT model, (b) PVA/XG-AgNO_3_@MMT. Figure S2: UHPLC-QTOF-MS/MS base peak chromatogram of *MRLE*. Figure S3: Trajectories of Ag particles in the crystal cell at 298 K: (a) PVA/XG-AgNPs, (b) PVA/XG-AgNPs@MMT, (c) PVA/XG-AgNO_3_@MMT. Figure S4: The titratable acid of (A) grapes and (B) bananas samples. Figure S5: The soluble solids content of (A) grape and (B) banana samples. Figure S6: The Vc content of grapes (A) and bananas (B). Figure S7: The pH of (A) grape and (B) banana samples. Table S1: Abbreviation and ratios of the composite films. Table S2: Experimental BBD with independent variables. Table S3: ANOVA results for the quadratic model. Table S4: Parameter for anneal dynamics. Table S5: Parameters for MD. References [56,57] are cited in the supplementary materials.
