# Supplementary material for: Green-Synthesized Silver Nanoparticle-Loaded Antimicrobial Films: Preparation, Characterization, and Food Preservation
Source: Foods. 2025 Jul 17;14(14):2509. doi: 10.3390/foods14142509 (PMC12294524; doi:10.3390/foods14142509)
Supplement: Supplementary file 1 [file foods-14-02509-s001.zip › foods-3724570-supplementary.pdf]

Supporting Information

Green-synthesized Silver Nanoparticle-Loaded Antimicrobial Films: Preparation, Characterization and Food Preservation

Wenxi Yu [1], Qin Lei [12], Jingxian Jiang [1], Jianwei Yan[2,*] , Xijian Yi [1], Juan Cheng [3], Siyu Ou [1], Wenjia Yin [4], Ziyan Li [1], and Yuru Liao [1]

1 School of Packaging Engineering, Hunan University of Technology, Zhuzhou, 412007, P. R. China; M230805Z1003@stu.hut.edu.cn
2 National Key Laboratory of Safety and Resilience of Civil Engineering in Mountain Area, East China Jiaotong University, Nanchang Jiangxi, 330013, PR China; jianwei@mail.ustc.edu.cn
3 College of Packaging Engineering, Jinan University, Zhuhai, 519070, P. R. China; chengj2008@jnu.edu.cn
4 School of packaging design and art, Hunan University of Technology, Zhuzhou, 412007, P. R. China; 13100235687@163.com
* Correspondence: jianwei@mail.ustc.edu.cn

Supporting Information S1.

Characterization of the films

Structural characterization of composite films: FTIR (Nicolet iS10, Thermo Fisher Scientific Inc.) in attenuated total reflection (ATR) mode was used to characterize the composite films. The spectra were recorded in the range of 4000-400 cm^{-1} with a scanning rate of 4 cm^{-1}. XRD patterns were recorded by using an X-ray diffractometer equipped (Thermo K-Alpha, Thermo Fisher Scientific Inc.) with a nickel-filtered Cu Kα radiation (λ=1.542 Å). The instrument was operated at 45 kV and 30 mA, scanning at a 2θ range of 5º to 40º with a step length of 2º and a scan speed of 2º/min. The surface morphology of the films was examined by using SEM (MIRA3LUM, Zeiss). Samples (10×10 mm^2) were analyzed at an acceleration voltage of 6 kV and 5000 magnification.

Mechanical properties of composite films: Mechanical testing was conducted based GB/T 1040.3-2006. Samples (100×10 mm^2) were vertically mounted in the grips of a universal testing machine (XLW(L)-P, Jinan Blu-ray Electromechanical Technology Co., LTD). Mechanical testing was performed at 25 °C and 50% relative humidity, with a crosshead speed of 50 mm/min as quasi-static state. Tensile strength (T_S) and elongation at break (E_{AB}) were determined by parallel experiment for 10 samples.

$$T_S = \frac{F}{S} \tag{1}$$

where F is the maximum tension force, and S is the cross-sectional area.

$$E_{AB} = \frac{(L - L_0)}{L_0} \times 100\% \tag{2}$$

Visible light transmittance : According to the method reported by Srinivasa et al. (Srinivasa, Ramesh, Kumar & Tharanathan, 2003), the standard background plate exhibited color values of L^* = 92.34, a^* = -1.66, and b^* = -4.55. The values of L^*, a^*, and b^* for the films were determined by using a colorimeter (10QC Guangdong Sanenshi Intelligent Technology Co., LTD). Each sample was measured 10 times and take the mean value. According to Han et al.'s method(HanFloros, 1997), the films were cut into 10×40 mm^2 strips and analyzed by using a UV-visible spectrophotometer (UV-5200, Shanghai Yuan Analysis Instrument Co., LTD) to measure transmittance at 280 nm and 600 nm for UV blocking and transparency evaluation.

Moisture absorption: Moisture absorption of the films was determined by using a static method, and the equilibrium moisture content was used to represent their hygroscopicity. Films of uniform thickness (20×40 mm^2) were cut and weighed their initial value (m_1). The pieces were placed in a constant temperature-humidity chamber at 25 °C and 75% RH. They were periodically weighed until the mass change was less than 0.1%, which states a moisture equilibrium. The final mass (m_2) of the samples was recorded, and equilibrium moisture content was calculated by

$$W_C = \frac{m_2 - m_1}{m_1} \times 100\% \tag{3}$$

Water solubility: Films (40×40 mm^2) were divided into 10 groups and immersed into 100 mL distilled water in beakers. After 6 h of soaking, the films were taken out and blotted dry with absorbent paper, and recorded it weight. The swelling degree was calculated as the ratio of mass increment of the piece to its initial mass.

Water vapor permeability: In accordance with the GB/T 26253-2010 standard, water vapor permeability (WVP) of the films was measured by using a WVP tester (C303M Jinan Languang Electromechanical Technology Development Company) under the condition of 23 ºC and 90% RH. Each sample was tested in triparallel experiment, and mean value was recorded

Postharvest preservation assay

Weight loss, hardness and pH: The weight of grapes from different batches were recorded on day 1, 2, 4, 6, and 8, and that of bananas were similarly recorded on day 1-5. The weight loss is calculated by

$$G = \frac{G_0 - G_m}{G_0} \times 100\% \tag{4}$$

where G_0 is the initial weight and Gm is the current weight. Fruit hardness was also measured by using a GY-4 hardness tester for grapes and bananas, while the pH was measured by using a digital pH meter.

Vitamin C(Vc) content and titratable acid(TA): Vc content was determined by homogenizing 20 g fruit pulp with 2% oxalic acid solution. A 2,6-dichlorophenolindophenol (DCIP) solution was prepared and mixed with Vc extract to observe the color change. Since *Vc* lead DCIP to translate from blue to colorless, its content could be calculated by the consumption of DCIP. TA of grapes and bananas was determined by acid-base titration. A 0.1 mol/L NaOH solution served as the standard for titrating the acidity of fruit sample. 10 g fruit sample was homogenized and diluted to 100 mL with distilled water. After a filtered process, 20 mL filtrate was titrated by using 0.01 mol/L NaOH. The solids content of grape and banana juice was measured by using a refractometer, and refractive index values were also recorded.

Supporting Information S2.

Molecular Dynamics

The molecular dynamics (MD) simulations were performed using the Forcite module. Initially, polymer chains (PVA and XG), Ag nanoparticles, and montmorillonite (MMT) layers were constructed. Each component was subjected to energy minimization using the Smart Minimizer method to ensure structural stability. Geometry optimization was then carried out using the Forcite–Geometry Optimization module until a stable potential energy state was reached.

Amorphous cell models containing Ag, MMT, XG, and PVA were built using the "Amorphous Cell" module. The simulations employed the Interface force field, with Ewald summation used for electrostatic interactions and atom-based calculations for van der Waals forces. Three composite systems were modeled: PVA/XG–AgNPs, PVA/XG–AgNPs@MMT, and PVA/XG–$AgNO_3$@MMT.

Each constructed cell was further optimized to eliminate potential energy overlaps. Annealing was performed using the Forcite–Anneal module, with parameters listed in Table S4. Subsequently, NVT ensemble dynamics simulations were run at 298 K for 5000 ps, as described in Table S5. After the simulations, analyses were conducted including mean square displacement (MSD) of Ag particles, diffusion trajectories, interaction energies between Ag, polymer, and MMT, as well as the free volume fraction within the composite structures.

Figures and Tables.

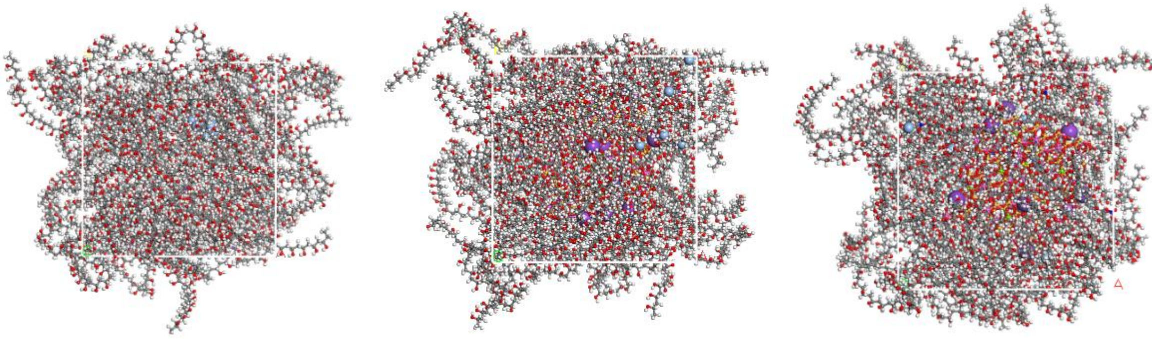

(a) (b) (c)

Figure S1 PVA/XG-AgNPs model (a), PVA/XG-AgNPs@MMT model (b), PVA/XG-AgNO3@MMT.

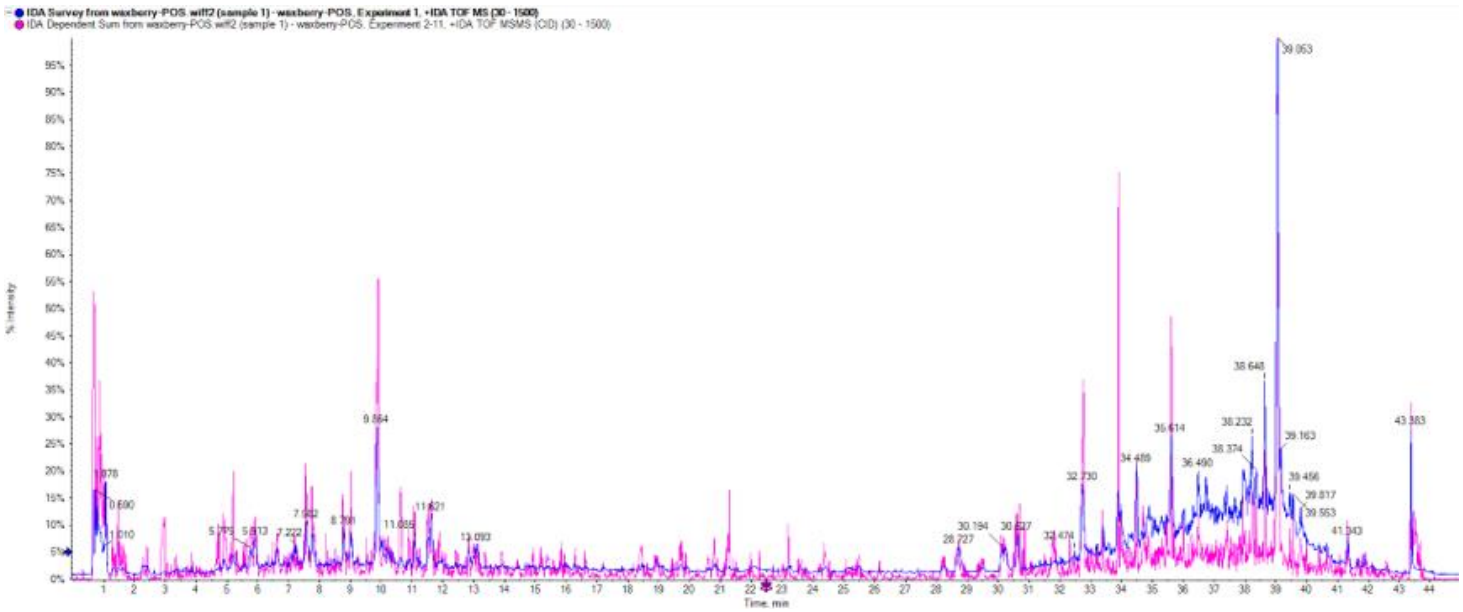

Figure S2 UHPLC-QTOF-MS/MS base peak chromatogram of *MRLE*.

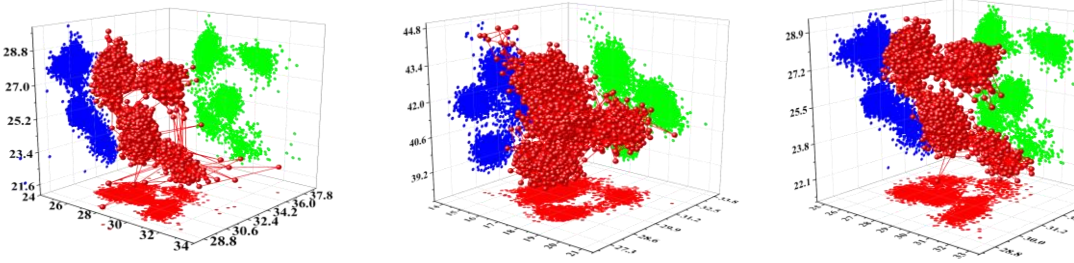

(a) (b) (c)

Figure S3 Trajectories of Ag particles in the crystal cell at 298 K (a) PVA/XG-AgNPs, (b) PVA/XG-AgNPs@MMT, (c) PVA/XG-AgNO3@MMT

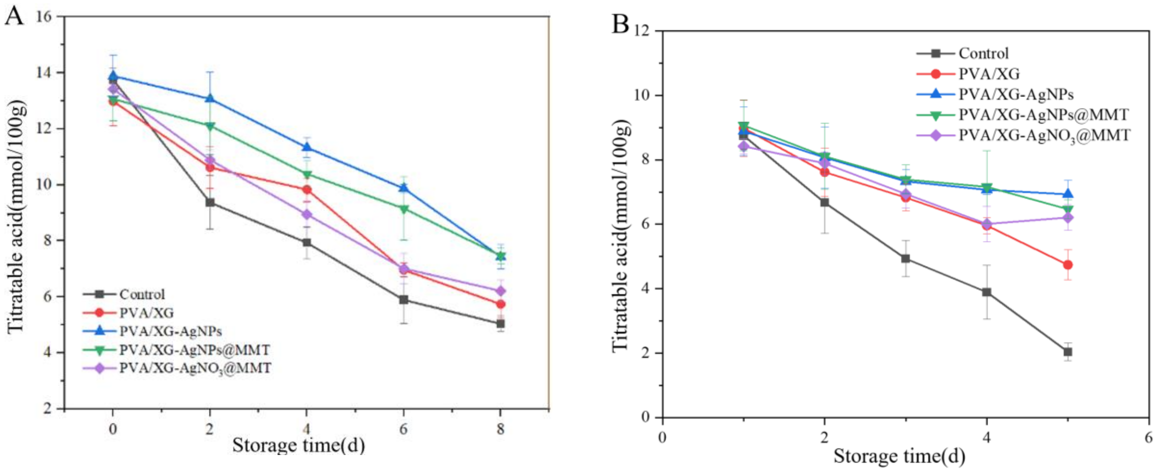

Figure S4. The titratable acid of (A) grapes and (B) bananas samples.

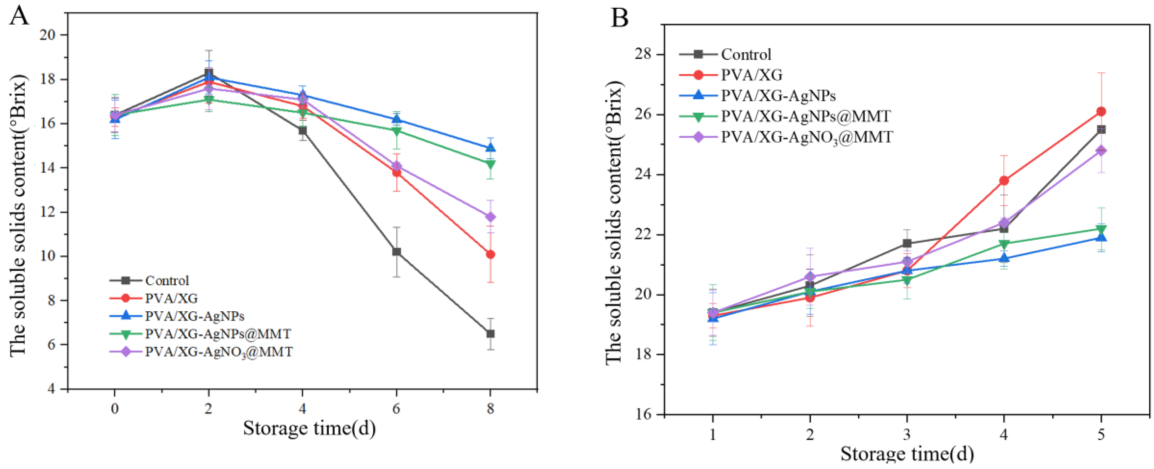

Figure S5. The soluble solids content of (A) grapes and (B) bananas samples

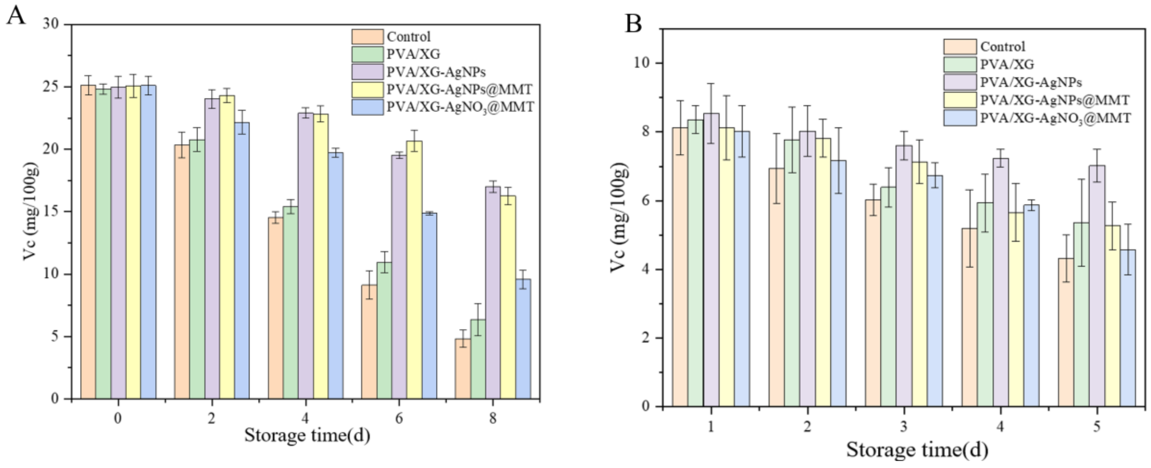

Figure S6. The Vc content of(A) grapes and (B)bananas.

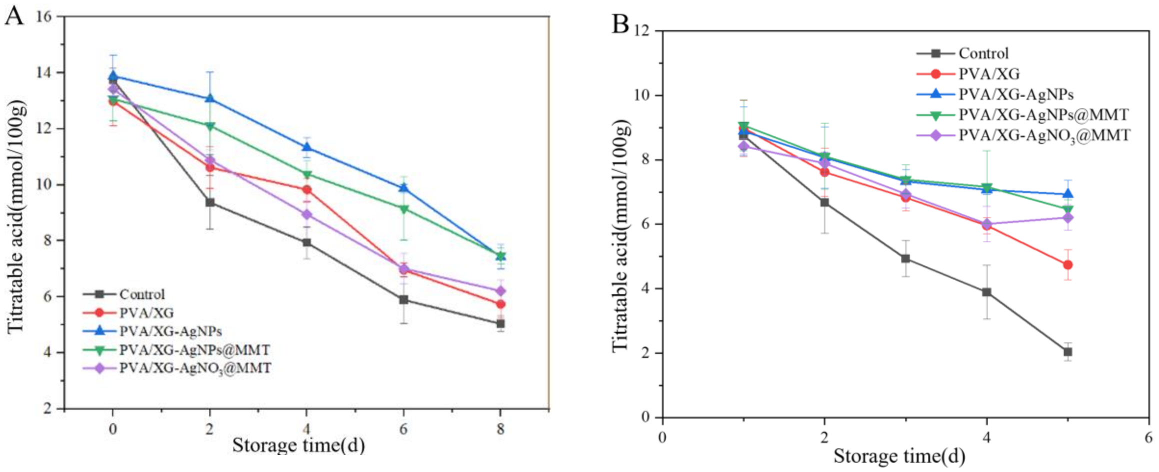

Figure S7. The pH of (A) grapes and (B) bananas samples.

Table S1 Abbreviation and ratios of the composite films

Film	PVA (g)	XG (g)	Ag (mg)	Ag/ mass fraction	MMT (g)
P	1.5	0.5	/	/	/
PA1	1.5	0.5	0.064	2%	/
PA2	1.5	0.5	0.108	4%	/
PA3	1.5	0.5	0.216	6%	/
PAM1	1.5	0.5	0.064	2%	0.03
PAM2	1.5	0.5	0.108	4%	0.03
PAM3	1.5	0.5	0.216	6%	0.03

PX: Pure PX film (control), PA1, PA2, PA3: PX film incorporated with 2%, 4%, and 6% AgNPs, respectively; PAM1, PAM2, PAM3: PX film incorporated with 2%, 4%, and 6% AgNPs@MMT, respectively.

Table S2 Experimental BBD with independent variables

Std Order	AgNO3 concentration (mMol/L)	Time (h)	Temperature (℃)	Absorbance
1	2	2	75	0.539
2	2.5	1.5	75	0.436
3	2	1.5	90	0.81
4	2	2	75	0.609
5	2	2.5	60	0.458
6	2.5	2	60	0.365
7	1.5	1.5	75	0.367
8	2	2	75	0.557
9	1.5	2	90	0.714
10	2.5	2	90	0.907
11	1.5	2.5	75	0.526

12	2	2	75	0.578
13	1.5	2	60	0.345
14	2.5	2.5	75	0.538
15	2	2	75	0.541
16	2	2.5	90	0.836
17	2	1.5	60	0.327

Table S3 ANOVA results for the quadratic model

Source	Sum of Squares	df	Mean Square	F-value	p-value	
Model	0.48	9	0.054	51.62	<0.0001	significant
A-AgNO3 concentration	0.011	1	0.011	10.41	0.0145	
B-Time	0.022	1	0.022	21.05	0.0025	
C-Temperature	0.39	1	0.39	378.27	<0.0001	
AB	8.123E-004	1	8.123E-004	0.78	0.4056	
AC	7.482E-003	1	7.482E-003	7.21	0.0313	
BC	2.756E-003	1	2.756E-003	2.66	0.1472	
A^2	0.016	1	0.016	15.36	0.0058	
B^2	5.617E-003	1	5.617E-003	5.41	0.0529	
C^2	0.027	1	0.027	25.63	0.0015	
Residual	7.263E-003	1	1.038E-003			
Lack of Fit	3.842E-003	1	1.281E-003	1.5	0.3434	not significant
Pure Error	3.421E-003	1	8.552E-004			
Cor Total	0.49	16				

Table S4 Parameter for Anneal Dynamics

Parameter	Settings	Parameter	Settings
Annealing cycle	5	The heating gradient for each cycle	5
Initial temperature	300K	The number of steps for each gradient simulation	10000
maximum temperature	600K	Force field	Interface

Table S5 Parameter for MD

Parameter	Settings	Parameter	Settings
Comprehensive Department	NVT	Temperature	298K
Temperature control method	Andersen	Sampling frequency	1 frame /2000 steps
Pressure control method	Berendsen	Sampling frequency	1fs
Force field	Interface		

References

Han, J.H., & Floros, J.D. (1997). Casting antimicrobial packaging films and measuring their physical properties and antimicrobial activity. Journal of Plastic Film & Sheeting 4 (13), 287-298.

Srinivasa, P.C., Ramesh, M.N., Kumar, K.R., & Tharanathan, R.N. (2003). Properties and sorption studies of chitosan – polyvinyl alcohol blend films. Carbohydrate Polymers 4 (53), 431-438.
